# Supplementary material for: A New In Vivo Zebrafish Bioassay Evaluating Liver Steatosis Identifies DDE as a Steatogenic Endocrine Disruptor, Partly through SCD1 Regulation
Source: Int J Mol Sci. 2023 Feb 15;24(4):3942. doi: 10.3390/ijms24043942 (PMC9959061; doi:10.3390/ijms24043942)
Supplement: Supplementary file 1 [file ijms-24-03942-s001.zip › s5_Supplementary Figure S1.pptx]

## Slide 1
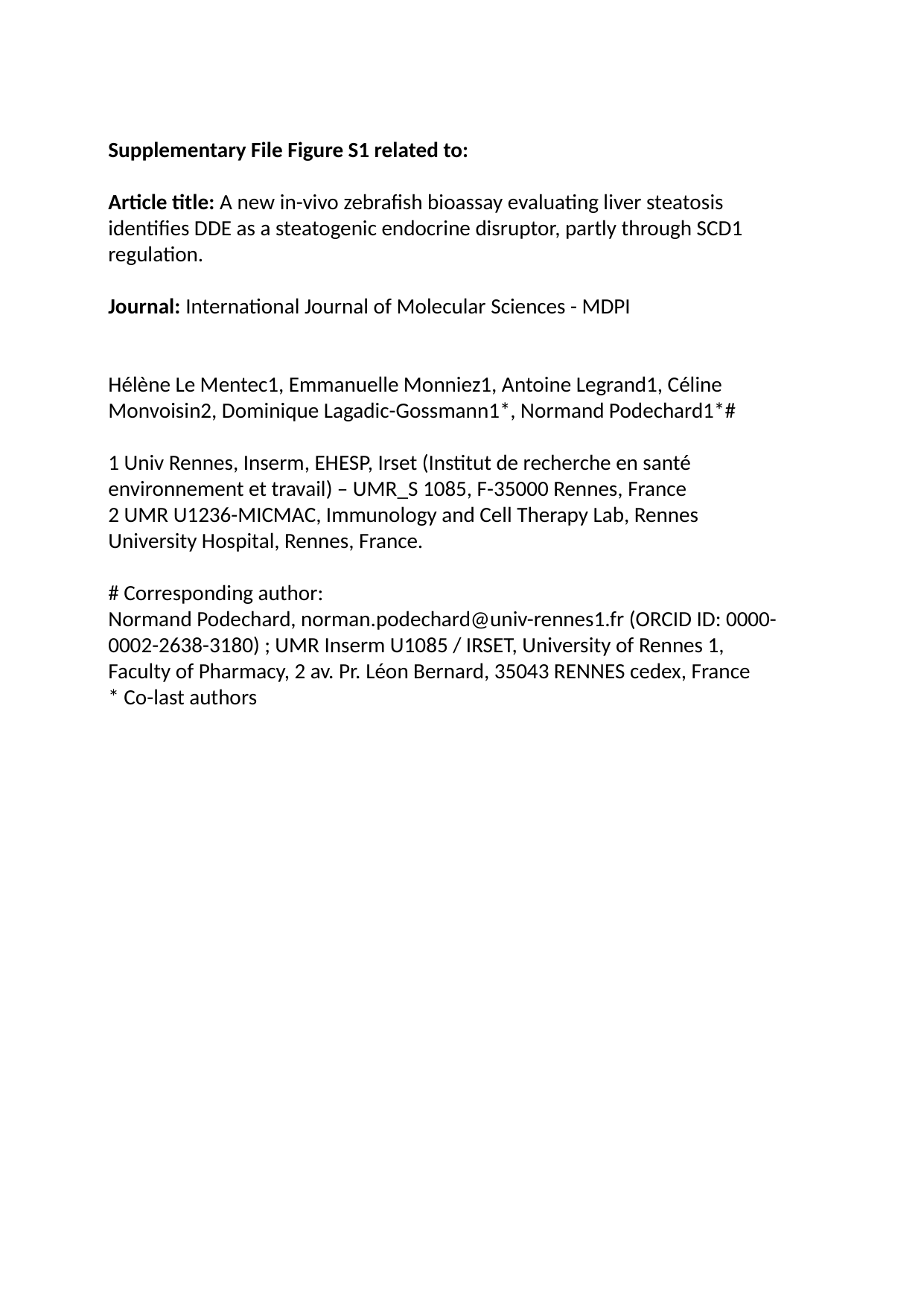

Supplementary File Figure S1 related to:
Article title: A new in-vivo zebrafish bioassay evaluating liver steatosis identifies DDE as a steatogenic endocrine disruptor, partly through SCD1 regulation.
Journal: International Journal of Molecular Sciences - MDPI
Hélène Le Mentec1, Emmanuelle Monniez1, Antoine Legrand1, Céline Monvoisin2, Dominique Lagadic-Gossmann1*, Normand Podechard1*#
1 Univ Rennes, Inserm, EHESP, Irset (Institut de recherche en santé environnement et travail) – UMR_S 1085, F-35000 Rennes, France
2 UMR U1236-MICMAC, Immunology and Cell Therapy Lab, Rennes University Hospital, Rennes, France.
# Corresponding author:
Normand Podechard, norman.podechard@univ-rennes1.fr (ORCID ID: 0000-0002-2638-3180) ; UMR Inserm U1085 / IRSET, University of Rennes 1, Faculty of Pharmacy, 2 av. Pr. Léon Bernard, 35043 RENNES cedex, France
* Co-last authors

## Slide 2
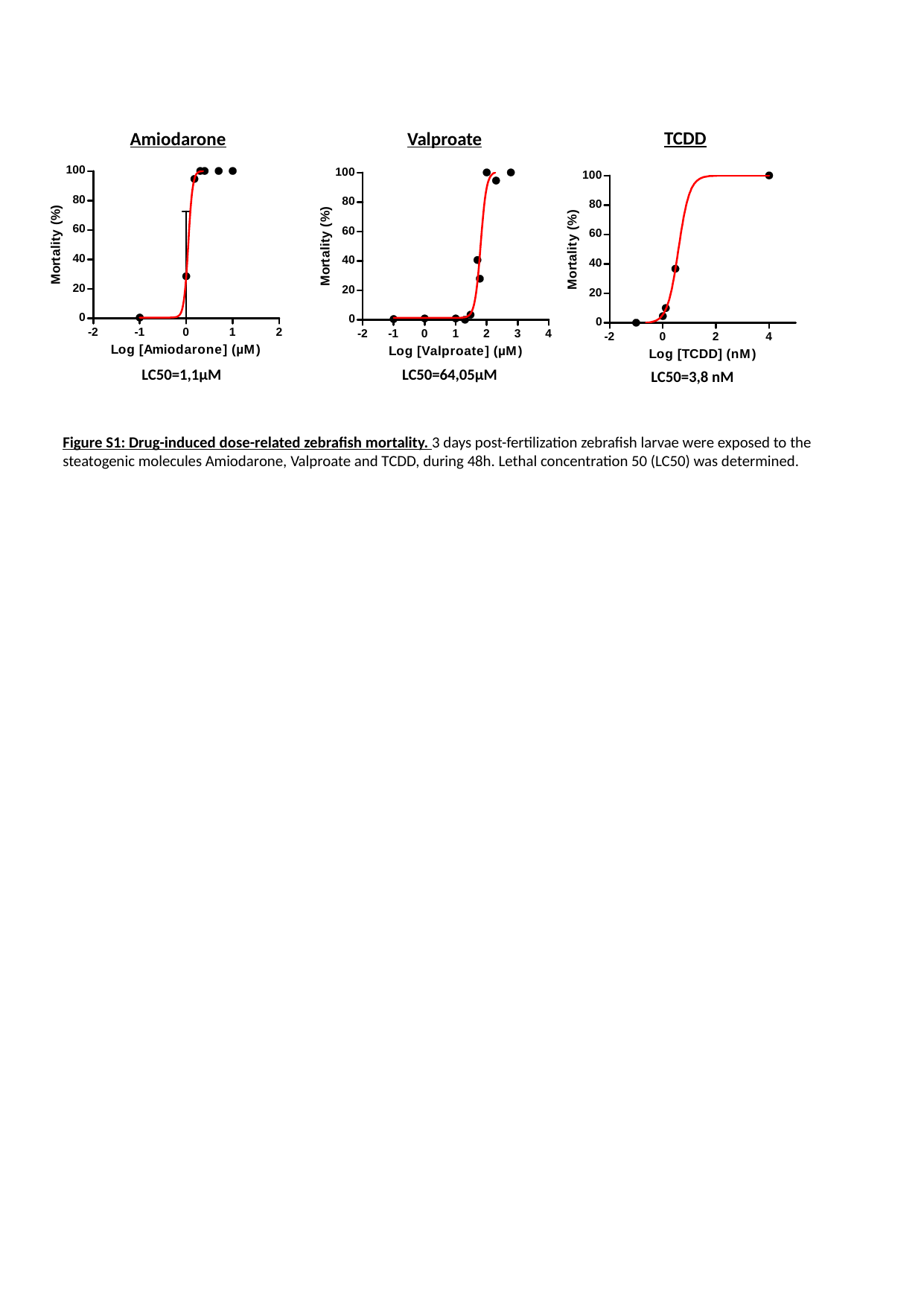

TCDD
Amiodarone
Valproate
LC50=1,1µM
LC50=64,05µM
LC50=3,8 nM
Figure S1: Drug-induced dose-related zebrafish mortality. 3 days post-fertilization zebrafish larvae were exposed to the steatogenic molecules Amiodarone, Valproate and TCDD, during 48h. Lethal concentration 50 (LC50) was determined.
