# Supplementary material for: A New In Vivo Zebrafish Bioassay Evaluating Liver Steatosis Identifies DDE as a Steatogenic Endocrine Disruptor, Partly through SCD1 Regulation
Source: Int J Mol Sci. 2023 Feb 15;24(4):3942. doi: 10.3390/ijms24043942 (PMC9959061; doi:10.3390/ijms24043942)
Supplement: Supplementary file 1 [file ijms-24-03942-s001.zip › s6_Supplementary Table S2.pdf]

Supplementary File Table S2 related to:

Article title: A new in-vivo zebrafish bioassay evaluating liver steatosis identifies DDE as a steatogenic endocrine disruptor, partly through SCD1 regulation.

Journal: International Journal of Molecular Sciences - MDPI

Hélène Le Mentec1, Emmanuelle Monniez1, Antoine Legrand1, Céline Monvoisin2, Dominique Lagadic-Gossmann1*, Normand Podechard1*#

1 Univ Rennes, Inserm, EHESP, Irset (Institut de recherche en santé environnement et travail) – UMR_S 1085, F-35000 Rennes, France

2 UMR U1236-MICMAC, Immunology and Cell Therapy Lab, Rennes University Hospital, Rennes, France.

# Corresponding author:

Normand Podechard, norman.podechard@univ-rennes1.fr (ORCID ID: 0000-0002-2638-3180) ; UMR Inserm U1085 / IRSET, University of Rennes 1, Faculty of Pharmacy, 2 av. Pr. Léon Bernard, 35043 RENNES cedex, France

* Co-last authors

Table S2: Selected chemical treatment studies in zebrafish. Comparison of the doses tested in the literature and those used for our studies

	Steatogenic molecules concentrations described in the literature to induce steatosis	Concentrations (µM) tested on the laboratory
**Amiodarone**	10-3.3-1.1 µM [1,2]	**1-0.1 µM**
**Valproïc acid**	600-200-67 µM [1, 3]	**60-10-1-0.1 µM**
**TCDD**	0.1-10 nM [5]<br>0-1000 ng/L (0-3 nM) [4]<br>0.01-100 µM [6]	**1-3 nM**
**Ethanol**	1-1.5-2% [4,7,8]	**1%**

References:

[1] Driessen M, Kienhuis AS, Pennings JL, Pronk TE, van de Brandhof EJ, Roodbergen M, Spaink HP, van de Water B, van der Ven LT. Exploring the zebrafish embryo as an alternative model for the evaluation of liver toxicity by histopathology and expression profiling. Arch Toxicol. 2013 May;87(5):807-23. doi: 10.1007/s00204-013-1039-z. Epub 2013 Apr 6. PMID: 23559145.

[2] Driessen M, Kienhuis AS, Vitins AP, Pennings JL, Pronk TE, van den Brandhof EJ, Roodbergen M, van de Water B, van der Ven LT. Gene expression markers in the zebrafish embryo reflect a hepatotoxic response in animal models and humans. Toxicol Lett. 2014 Oct 1;230(1):48-56. doi: 10.1016/j.toxlet.2014.06.844. Epub 2014 Jul 24. PMID: 25064622.

[3] He JH, Guo SY, Zhu F, Zhu JJ, Chen YX, Huang CJ, Gao JM, Dong QX, Xuan YX, Li CQ. A zebrafish phenotypic assay for assessing drug-induced hepatotoxicity. J Pharmacol Toxicol Methods. 2013 Jan-Feb;67(1):25-32. doi: 10.1016/j.vascn.2012.10.003. Epub 2012 Nov 3. PMID: 23128142.

[4] Zhang X, Li C, Gong Z. Development of a convenient in vivo hepatotoxin assay using a transgenic zebrafish line with liver-specific DsRed expression. PLoS One. 2014 Mar 13;9(3):e91874. doi: 10.1371/journal.pone.0091874. PMID: 24626481; PMCID: PMC3953600.

[5] Li ZH, Xu H, Zheng W, Lam SH, Gong Z. RNA-sequencing analysis of TCDD-induced responses in zebrafish liver reveals high relatedness to in vivo mammalian models and conserved biological pathways. PLoS One. 2013 Oct 30;8(10):e77292. doi: 10.1371/journal.pone.0077292. PMID: 24204792; PMCID: PMC3813628.

[6] Ton C, Lin Y, Willett C. Zebrafish as a model for developmental neurotoxicity testing. Birth Defects Res A Clin Mol Teratol. 2006 Jul;76(7):553-67. doi: 10.1002/bdra.20281. PMID: 16933308.

[7] Passeri MJ, Cinaroglu A, Gao C, Sadler KC. Hepatic steatosis in response to acute alcohol exposure in zebrafish requires sterol regulatory element binding protein activation. Hepatology. 2009 Feb;49(2):443-52. doi: 10.1002/hep.22667. PMID: 19127516; PMCID: PMC2635426.

[8] Kashyap B, Pegorsch L, Frey RA, Sun C, Shelden EA, Stenkamp DL. Eye-specific gene expression following embryonic ethanol exposure in zebrafish: roles for heat shock factor 1. Reprod Toxicol. 2014 Jan;43:111-24. doi: 10.1016/j.reprotox.2013.12.002. Epub 2013 Dec 16. PMID: 24355176; PMCID: PMC3918430.
