# Supplementary material for: A New In Vivo Zebrafish Bioassay Evaluating Liver Steatosis Identifies DDE as a Steatogenic Endocrine Disruptor, Partly through SCD1 Regulation
Source: Int J Mol Sci. 2023 Feb 15;24(4):3942. doi: 10.3390/ijms24043942 (PMC9959061; doi:10.3390/ijms24043942)
Supplement: Supplementary file 1 [file ijms-24-03942-s001.zip › s7_Supplementary Figure S2.pptx]

## Slide 1
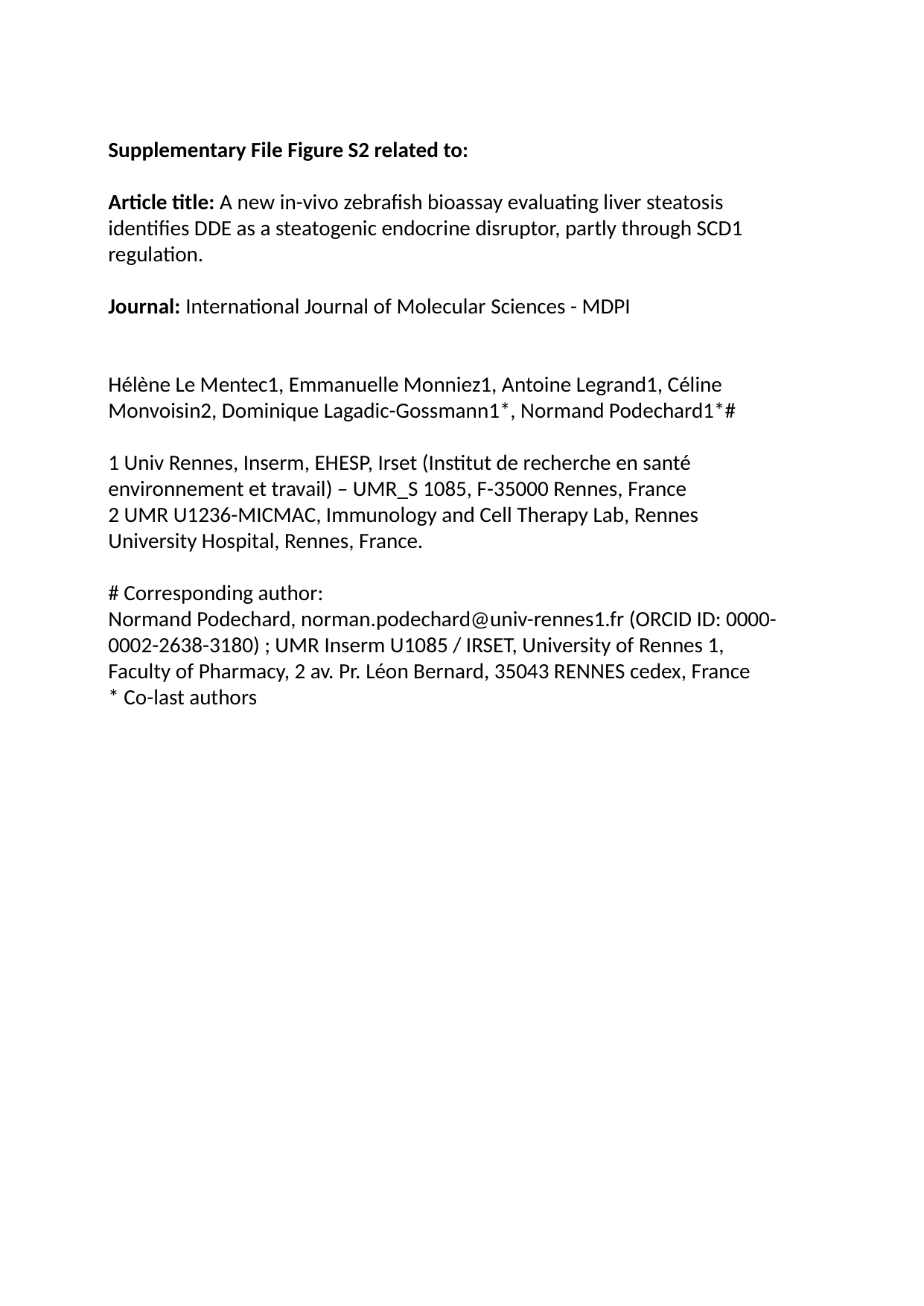

Supplementary File Figure S2 related to:
Article title: A new in-vivo zebrafish bioassay evaluating liver steatosis identifies DDE as a steatogenic endocrine disruptor, partly through SCD1 regulation.
Journal: International Journal of Molecular Sciences - MDPI
Hélène Le Mentec1, Emmanuelle Monniez1, Antoine Legrand1, Céline Monvoisin2, Dominique Lagadic-Gossmann1*, Normand Podechard1*#
1 Univ Rennes, Inserm, EHESP, Irset (Institut de recherche en santé environnement et travail) – UMR_S 1085, F-35000 Rennes, France
2 UMR U1236-MICMAC, Immunology and Cell Therapy Lab, Rennes University Hospital, Rennes, France.
# Corresponding author:
Normand Podechard, norman.podechard@univ-rennes1.fr (ORCID ID: 0000-0002-2638-3180) ; UMR Inserm U1085 / IRSET, University of Rennes 1, Faculty of Pharmacy, 2 av. Pr. Léon Bernard, 35043 RENNES cedex, France
* Co-last authors

## Slide 2
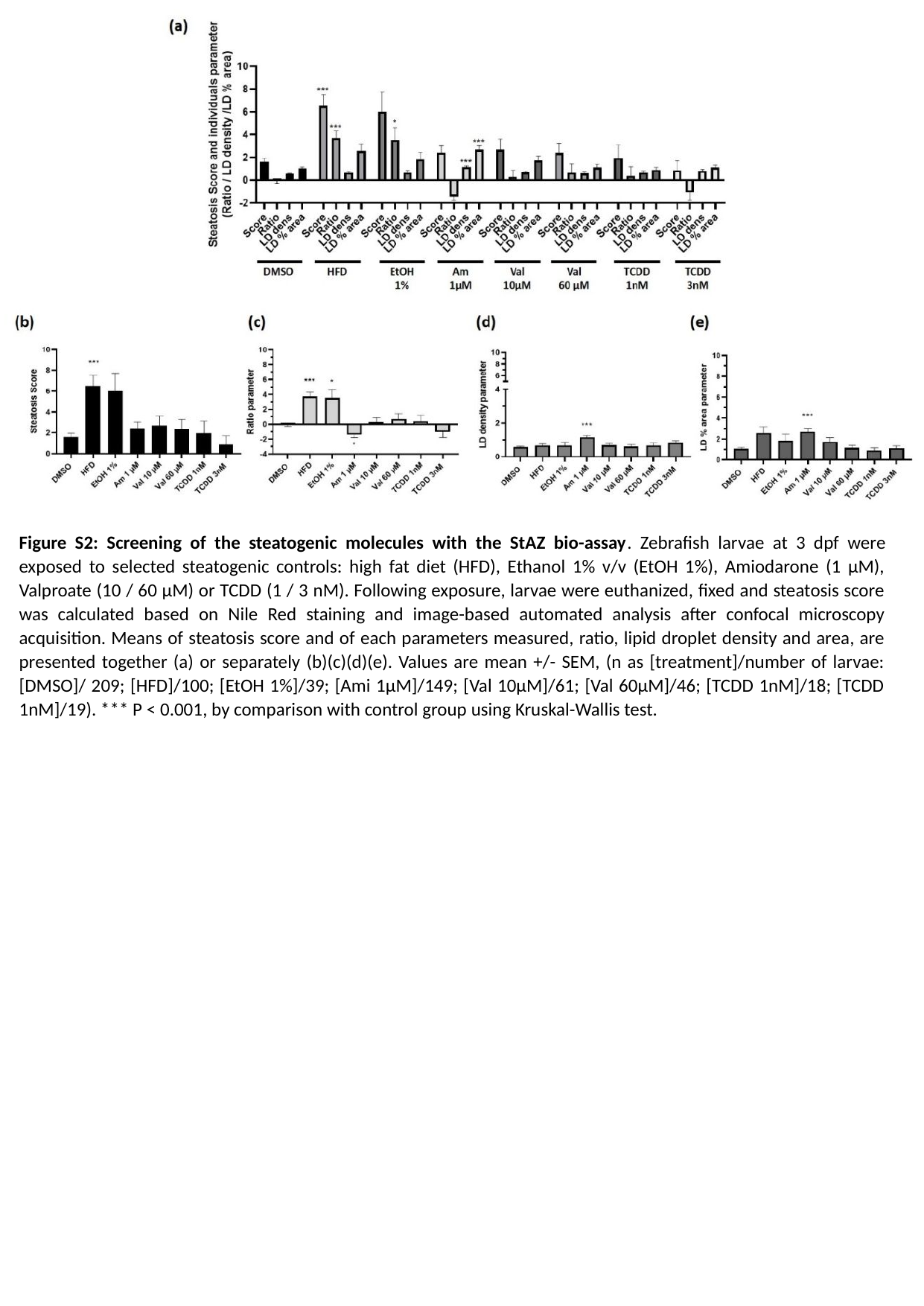

Figure S2: Screening of the steatogenic molecules with the StAZ bio-assay. Zebrafish larvae at 3 dpf were exposed to selected steatogenic controls: high fat diet (HFD), Ethanol 1% v/v (EtOH 1%), Amiodarone (1 µM), Valproate (10 / 60 µM) or TCDD (1 / 3 nM). Following exposure, larvae were euthanized, fixed and steatosis score was calculated based on Nile Red staining and image-based automated analysis after confocal microscopy acquisition. Means of steatosis score and of each parameters measured, ratio, lipid droplet density and area, are presented together (a) or separately (b)(c)(d)(e). Values are mean +/- SEM, (n as [treatment]/number of larvae: [DMSO]/ 209; [HFD]/100; [EtOH 1%]/39; [Ami 1µM]/149; [Val 10µM]/61; [Val 60µM]/46; [TCDD 1nM]/18; [TCDD 1nM]/19). *** P < 0.001, by comparison with control group using Kruskal-Wallis test.
